# Endophytic Effects of *Beauveria bassiana* on Corn (*Zea mays*) and Its Herbivore, *Rachiplusia nu* (Lepidoptera: Noctuidae)

**DOI:** 10.3390/insects10040110

**Published:** 2019-04-18

**Authors:** María Leticia Russo, Ana Clara Scorsetti, María Florencia Vianna, Marta Cabello, Natalia Ferreri, Sebastian Pelizza

**Affiliations:** 1 Instituto de Botánica Carlos Spegazzini, Facultad de Ciencias Naturales y Museo, Universidad Nacional de La Plata- CICPBA Calle 53 # 477, La Plata 1900, Argentina; leticiarusso@conicet.gov.ar (M.L.R.); ascorsetti@conicet.gov.ar (A.C.S.); mcabello@ymail.com (M.C.); nati_f@live.com.ar (N.F.); sebastianpelizza@conicet.gov.ar (S.P.); 2 Comisión de Investigaciones Científicas de la Provincia de Buenos Aires (CICPBA), Calle 526 entre 10 y 11, La Plata 1900, Argentina

**Keywords:** endophytic *Beauveria bassiana*, *Zea mays*, *Rachiplusia nu*, growth parameters, food preference

## Abstract

Entomopathogenic fungi are widely recognized as agents of biological control worldwide. Their use in agriculture for the regulation of pest populations is a promising alternative to conventional insecticides. Furthermore, recent studies have shown that entomopathogenic fungi fulfill an additional role in plants as growth promoters. The purpose of this investigation was to assess the growth and yield of corn plants colonized with *Beauveria bassiana* and its effect on the lepidopteran pest *Rachiplusia nu.* Effects of the fungus on plant growth, crop yield, and vertical transmission were evaluated in the field. Feeding preferences of *R. nu* larvae were assessed in the laboratory using a “choice test”. Corn plants inoculated with *B. bassiana* showed an increase in height, number of leaves, grain weight, yield, and percentage of seed germination compared to control plants. Consumption of *B. bassiana*-colonized corn plants by *R. nu* larvae was reduced compared to feeding levels observed on non-inoculated plants. This study showed that endophytic *B. bassiana* can provide multiple benefits to *Zea mays* and can play an important role in future integrated pest management programs.

## 1. Introduction

Corn (*Zea mays* L.) is a member of the Poaceae family and it is originally from the Americas, particularly from Mexico, Central America, and South America. In this continent, it is the coarse grain with the largest area planted and the largest volume of production in annual tons. The primary economy of Argentina is mainly based on the cultivation of corn and Argentina is one of the five largest producers of corn worldwide [1,2]. Chemical fertilizers represent 30% of the crop production costs, and each year the amount of fertilizers and pesticides used increases in order to promote productivity, and as the land area in production grows. Arthropod pests restrict the production and quality of the grains of this crop, generating a gap between the annual average yields and the crop potential. Among the main corn pests are the lepidopteran defoliators. *Rachiplusia nu* (Guennée) is widely distributed in South America and is one of the primary lepidopteran pests of corn in this region [3]. Although chemical insecticides are effective against most pests, their indiscriminate use has generated a series of ecological problems, such as the development of resistance, environmental contamination, and negative impacts on human health [4,5]. The use of entomopathogenic fungi as endophytes is an alternative to chemical insecticides but may also be used to supplement or reduce the use of fertilizers. The fungi can act as plant growth-promoting agents, exhibiting important changes in the growth and yield of several crops [6,7,8,9,10,11,12,13,14,15,16].

The entomopathogenic fungus *Beauveria bassiana* (Bals. -Criv.) Vuill. has been endophytically introduced with success in different plant species and has shown activity against different pests [17]. In corn, its insecticidal capacity was demonstrated against the lepidopterans *Sesamia calamistis* Hampson [18], *Ostrinia nubilalis* (Hübner 1796) [19], and *Spodoptera frugiperda* Smith. [20]. Based on the previously mentioned background information and considering the potential benefits of entomopathogenic fungi as endophytes in plants, the purpose of this investigation was to assess the growth and yield of maize plants endophytically colonized by *B. bassiana* and its effect on the lepidopteran pest *R. nu*.

## 2. Materials and Methods

Two experiments were performed to evaluate the potential of endophytic *B. bassiana* introduced into corn plants by the leaf spraying technique [21]. In each experiment, we compared two treatments, plants inoculated with *B. bassiana* vs. non-inoculated plants. The first experiment was conducted during the years 2013–2014 (November–March) and 2014–2015 (November–March) in agreement with the crop cycle. The aim of this experiment was to analyze the effect of the entomopathogenic fungi on different growth parameters and the performance of these plants under field conditions. In a second experiment, performed in the laboratory, the food preference of *R. nu* was evaluated.

Corn plants were grown from seeds of the hybrid DK 699 MG (DEKALB-Monsanto, St. Louis, MO, USA). The seeds’ surfaces were sterilized by successive immersion in 2% sodium hypochlorite for two minutes, then 70% alcohol for three minutes, and finally three washes in sterile distilled water were made. The water from the last rinse was inoculated onto Petri dishes containing potato dextrose agar (PDA, Britania^®^ S.A., Buenos Aires, Argentina) to evaluate if the sterilization method was effective. The seeds were dried on filter paper and left at 4 °C overnight to synchronize the growth [22] and thereafter were sown into 500 cm^3^ pots with sterile soil, perlite, and vermiculite (1:1:1). The pots were maintained under controlled conditions (25 °C and 12/12 h L:D) and water was provided as needed.

*B. bassiana* LPSc 1098 (GenBank KT163259) was acquired from the mycological collection of “Instituto de Botánica Spegazzini” (Buenos Aires, Argentina). The strain was selected due to its high sporulation capacity [23] and high rate of plant colonization as well as its proven efficacy against insect pests, causing the larval mortality of *Helicoverpa gelotopoeon* (Dyar), and *S. frugiperda* in laboratory bioassays [24]. To obtain the conidial suspension, the strain LPSc 1098 *B. bassiana* was cultivated onto PDA and incubated at 25 °C in darkness. After 15 days, conidia were harvested by scrapping them off the Petri dishes and were transferred to 10 mL of 0.01% (*v*/*v*) Tween 80 (polyoxyethylene sorbitan monolaurate) (Merck, Kenilworth, NJ, USA). The suspension was filtered and homogenized by shaking for 10 min. Conidial concentration was determined by using a Neubauer chamber and adjusted to 1 × 10^8^ conidia/mL [21]. Conidial viability was assessed prior to inoculation of plants by plating 50 µL of 1 × 10^8^ conidia/mL solution on PDA and incubating at 25° C for 18 h. Three random groups of 100 conidia were examined using a microscope to estimate percent germination. A conidium was considered to have germinated when a visible germ tube longer than half the diameter of the conidium was observed. Conidial germination was 95% and was considered acceptable for use in the experiments [25].

Plants were inoculated by the leaf spraying technique, using a glass hand sprayer (30 mL capacity) to spray each seedling with 3 mL of conidial suspension.

The insects were acquired from AgIdea (www.agidea.com.ar, Buenos Aires, Argentina), and bred for two generations in a bioterium under controlled conditions for temperature, relative humidity, and photoperiod (25 ± 2 °C, 70–75% RH, and 14:10 h L:D, respectively), according to Greene et al. [26]. Larvae were placed individually in Petri dishes with artificial diet as food source, which consisted of bean flour, beer yeast, methylparaben, ascorbic and sorbic acids, streptomycin, formaldehyde, vitamin complex, agar, and distilled water. Pupae were sexed and placed in couples in plastic containers (500 cm^3^). Adults were fed with a 10 % sugar solution until they laid eggs. These were reared until third instar larvae, which were then utilized in the bioassay.

### 2.1. Experiment I

The first experiment was carried out in the town of Alberti (35°01′00″ S–60°16′00″ W), Buenos Aires, Argentina, where corn is one of the main crops. In this region, the annual precipitation is 1050 mm and the temperature range over the crop cycle is 10–32 °C with an average of 20 °C [27].

The trial was performed with two treatments of 40 plants each and performed over two consecutive years, thus totaling 80 plants per treatment. Corn seedlings were first grown in a greenhouse at 25 °C and a 12:12 h L:D photoperiod for two weeks. To prevent deactivation of the conidia by exposure to UV rays, plants were inoculated inside the greenhouse using the leaf spraying method. Previous studies proved that leaf spraying is the most effective method for this *B. bassiana* strain to colonize corn plants [21]. Control plants were inoculated using the same methodology, but sprayed with a 0.01% solution of Tween 80 only. Two weeks after treatment, both the plants inoculated with the fungus and the control plants were transplanted to the field. Plants were distributed in a randomized way in rows (spacing between rows of 0.75 m) and in each row 10 plants were planted 0.50 m apart from each other.

Prior to transplanting the plants in the field, in order to asses endophytic colonization one leaf from each of the 40 plants was randomly selected for each treatment. These were washed twice in distilled water under laminar flow, then surface-sterilized by immersion for 1 min in 70% (*v*/*v*) ethanol, then 4 min in sodium hypochlorite (3%, *v*/*v* available chlorine), and finally washed three times in sterilized distilled water. After surface sterilization, samples were cut into 1-cm^2^ pieces with a sterile scalpel and aseptically transferred to plates containing 20 mL of PDA medium with 2 mL of antibiotics (0.5 g streptomycin and 0.25 g chloramphenicol/200 mL). The presence of *B. bassiana* in inoculated plants and the absence in control plants was corroborated after incubating plates at 25 °C in darkness for 72 h. The water from the last rinse was plated onto PDA media and incubated for 10 days at 25 °C to determine whether the sterilization process was successful in eliminating epiphytic micro-organisms. If we observed fungal growth, we did not consider the corresponding samples for analyses [21].

#### 2.1.1. Measurement of Growth Parameters and Crop Yield

The effects the *B. Bassiana* LPSc 1098 as an endophyte were assessed using methods described by Distéfano et al. [28] and Lauer [29] when corn plants finished their cycle (five months). Total height, number of leaves, height and node number where the first cob emerged, number of rows per cob and number of grains per row, weight of grains per plant, number of cobs per plant, and crop yield were evaluated.

#### 2.1.2. Vertical Transmission

To evaluate the potential for vertical transmission (from plant to seeds), 10 seeds from each inoculated plant (*n* = 800) and 10 seeds of each control plant (*n* = 800) were surface-sterilized as was previously explained for the seeds utilized at the beginning of the experiment [30], cut and placed onto Petri dishes containing a selective medium for entomopathogenic fungi that consisted of oatmeal agar with 0.5 g/L chloramphenicol as basal medium amended with 0.6 g/L CTAB (cetyltrimethyl ammonium bromide, Britania^®^, Buenos Aires, Argentina) [31]. Dishes were incubated in a chamber at 24 °C in darkness for 10 days to corroborate the presence/absence of the endophyte emerging from the seeds.

In addition, another 10 seeds from each inoculated plant (*n* = 800) and 10 seeds from each control plant (*n* = 800) were sown into pots with sterile substrate of soil, perlite, and vermiculite (1:1:1). This substrate was sterilized in an autoclave for 45 min at 121 °C, which was performed three times with 24-h intervals between each process [32]. The pots were maintained in a greenhouse under controlled conditions for temperature, humidity, and photoperiod (24 °C, 75% RH, 14:10 h L:D, respectively). To confirm the presence of the fungus as an endophyte, one leaf was removed from plants grown from seeds obtained from inoculated plants and from plants grown from seeds of control plants. Leaves surfaces were sterilized under the same conditions that were mentioned at the beginning of Experiment I. Four leaf fragments (10 × 10 mm) were placed onto sterile filter paper and allowed to dry under sterile conditions in a laminar flow cabinet. After drying, the fragments were placed onto Petri dishes containing PDA culture medium plus antibiotics (0.5 g streptomycin and 0.25 g chloramphenicol) and were held at 25 °C in the dark for 10 days in order to corroborate the presence/absence of the endophyte emerging from the leaves.

#### 2.1.3. Percentage of Seed Germination

The percentage of seed germination from both inoculated and control plants was determined using two samples of 100 seeds each per treatment, and germination was assessed using methods modified from those described by Luna and Iannone [33], where the sample size was different and adhering to the standards of the “International Association of Seed Analysis” [34]. Sterilized and moistened sand was used as germination substrate. Seed germination was assessed in a chamber at 25 °C and a 12:12 h L:D photoperiod. The number of non-germinated and normal and abnormal seedlings was recorded 8 days after sowing. The percentage of seed germination was calculated as the percentage (%) of seeds that germinated and produced normal seedlings at the end of the test period.

### 2.2. Experiment II

#### Feeding Preference

To study the feeding preference of *R. nu* larvae for *B. bassiana* colonized vs. control (non-colonized) corn leaves, a “choice test” [35] was carried out. Inoculated and not-inoculated (control) leaves were simultaneously provided to the insects. The endophytic colonization by the entomopathogen was confirmed as in Experiment I. Fragments of 4 × 2 cm from 21-day-old leaves from inoculated and control plants were scanned to determine their initial surface area. Two leaf fragments, one from plants colonized with *B. bassiana* and the other from non-colonized plants that were used as controls, were placed into Petri dishes (90 mm diam.) over moistened filter paper. One L2 larva was placed inside each Petri dish and left for 24 h. Three independent replicates of 30 individuals each were set up. At the end of the experiment, the leaf fragments were scanned again utilizing ImageJ software [36] to determine the final area eaten (initial area—area after consumption) by the larvae, according to the methods of Crawford et al. [35].

### 2.3. Data Analysis

Dietary preference, percentage of seed germination, crop yield, and each of the growth parameters were compared between plants inoculated with *B. bassiana* and controls using Student’s *t*-test utilizing InfoStat software [37].

## 3. Results

### 3.1. Experiment I—Growth and Crop Yield in Plants Inoculated with B. bassiana

The endophytic association of the entomopathogenic fungus *B. bassiana* with corn plants promoted yield and plant growth. As shown in Table 1, the following morphological and reproductive parameters increased significantly in inoculated plants: plant height (t = 8.63, df = 158, *p* < 0.0001), number of leaves (t = 12.05, df = 158, *p* < 0.0001), height where the first cob emerged (t = 5.99, df = 158, *p* <0.0001), node number where the first cob emerged (t = 7.02, df = 158, *p* < 0.0001), number of rows per cob (t = 10.39, df = 158, *p* < 0.0001), number of grains per row (t = 8.35, df = 158, *p* < 0.0001), number of cobs per plant (t= 9.63.; df = 158, *p* < 0.0001), seed weight per plant (t = 9.64, df = 158, *p* < 0.0001), and crop yield (t = 8.39, df = 10, *p* < 0.0001).

#### Vertical Transmission of *B. bassiana* and Percentage of Seed Germination

Our results showed that *B. bassiana* LPSc 1098 was not transmitted vertically from the parental generation to the seeds. The entomopathogenic fungus was neither isolated from the seeds obtained from the plants grown in the field nor from the seedlings from those seeds.

However, the percentage of seed germination was significantly different between treatments (t = 5.85, df = 6, *p* < 0.0011) (Table 1).

### 3.2. Experiment II

#### Feeding Preference

**Figure 1:**
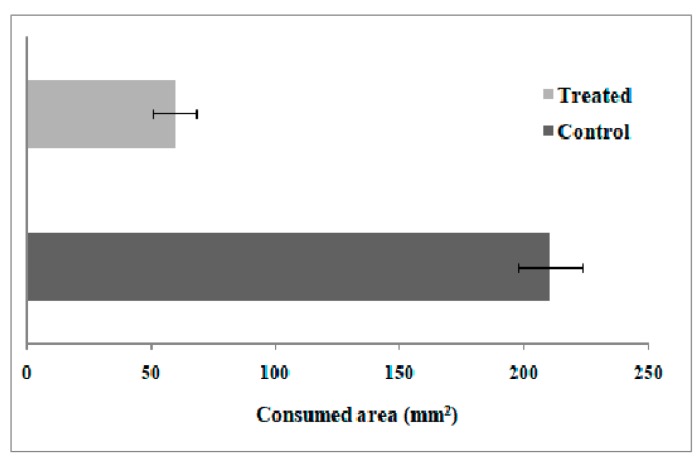
Total area consumed (mm^2^) by *Rachiplusia nu* larvae fed with *Beauveria bassiana*-colonized corn plants (treated) and non-colonized corn plants (control).

Endophytic colonization of corn leaves by *B. bassiana* affected the leaf area consumed by *R. nu* larvae. As shown in Figure 1, significant differences in leaf area consumed (t = 5.23, df = 88, *p* < 0.0001) were found between inoculated and non-inoculated (control) plants.

## 4. Discussion

Our study demonstrated the positive effects of endophytic *B. bassiana* LPSc 1098 on the growth and yield of corn plants grown under field conditions. Similar effects with the same endophytic strain have also been reported on the growth parameters of different plant species [16]. Castillo López and Sword [10] demonstrated an increase in the height of *Gossypium hirsutum* plants and in the size of their fruits as a result of endophytic colonization by *B. bassiana*. Similarly, Sanchez Rodriguez et al. [12], using the seed immersion and soil inoculation techniques, showed that *B. bassiana* increased the growth and development of wheat plants, as well as the weight of the ears and the length of the roots. In contrast to our results, these authors found that *B. bassiana* caused a decrease in the weight of grains/plant compared to control plants. Our findings concur with those of Jaber and Enkerli [13] and Dash et al. [15], who found that the endophytic *B. bassiana* stimulated plant growth and led to an increase in the number of leaves produced by *Vicia faba* and *Phaseolus vulgaris*, respectively. Conversely, Akello et al. [38] showed that the presence of the fungus did not affect the growth of banana, and Qayyum et al. [39] observed a decrease in the size of tomato fruits harvested from plants colonized by the fungus. Our results concur with those of Kabaluk and Ericsson [6] and Sanchez Rodriguez et al. [12], who observed a significant increase in crop yield of corn and wheat, respectively, from plants colonized by entomopathogenic fungi. These divergent results might be due to the strong endophytic association of specific fungal endophytes strains with host plant species and even plant species varieties [13,40].

Regarding the mechanisms related to plant growth promotion, previous studies suggested that *B. bassiana* could reduce the damage caused by insect pests and/or act as an antagonist against certain pathogens [7,41]. The production of plant growth regulators by endophytic fungi is believed to promote plants growth and yield. There are several studies regarding auxins and cytokinins, two main hormones present in plants, that have been proven to be synthesized by entomopathogenic fungi endophytes [42]. In this sense, Liao et al. [42] showed that *Metarhizium robertsii* colonizing *Arabidopsis* sp. plants produces indole-3-acetic acid (IAA), which stimulates cellular division. Furthermore, previous studies by Joseph et al. [43] and Jirakkakul et al. [44] evidenced the production of structures called siderophores, which can modify the bioavailability of nutrients in the plant and thereby stimulate growth.

Related with percentage of seed germination obtained from inoculated plants, we observed an increase in this parameter in the plants that were inoculated with *B. bassiana* LPSc (1098) strain as endophyte. Although the vertical transmission of endophytic fungal entomopathogens has been demonstrated by only a few studies [45], we could not observe the vertical transmission of *B. bassiana* LPSc (1098) strain. These results may seem contradictory, but it can be hypothesized that the percentage of seed germination could not be influenced directly by *B. bassiana*, and may instead be an indirect effect of the presence of the fungus in the parent plant. Mechanisms underlying these observations need to be further investigated. Our results showed that *R. nu* larvae consumed less of corn leaves endophytically-colonized by *B. bassiana*. Martinuz et al. [45], using a choice test, also demonstrated that aphids preferred to feed on plants not colonized with the entomopathogenic fungi *Fusarium oxysporum*. Similar results were obtained for *H. zea*, *H. gelotopoeon*, and *S. calamistis* [10,24,46]. In contrast, Vianna et al. [47] concluded that *H. gelotopoeon* larvae preferentially fed on inoculated rather than non-inoculated tobacco leaves. There have been several attempts to explain the reduction in the consumption by insects when feeding on inoculated plants. It has been proposed that the production of secondary metabolites, the production of superoxides, changes in the phytosterol profile of plants, or the induction of an indirect systemic response could be responsible for this change of behavior in insects [10]. In this sense, the investigation performed by Shrivastava et al. [48] demonstrated that plants inoculated with *B. bassiana* showed higher levels of terpenoids, which are considered secondary metabolites with antiherbivore properties. These studies highlight the importance of incorporating the use of entomopathogenic fungi endophytes into strategies for protecting plants from pests and enhancing plant growth.

## 5. Conclusions

This study broadens our knowledge on the potential of entomopathogenic fungi as endophytes in corn plants. The presence of *B. bassiana* LPSc 1098 as an endophyte in maize plants negatively affected the feeding preference of inoculated leaves by *R. nu* and resulted in an increase in plant growth and yield. In this context, these microorganisms could be used in integrated pest management programs, which in turn could lead to reductions in the use of agrochemicals.

## Figures and Tables

**Table 1:** Growth parameters, yield characteristics, and seed germination from corn plants colonized with *Beauveria bassiana* (LPSc 1098) (treated) and non-colonized corn plants (control). Mean values ± SEM presented. Within each parameter measured (row) numbers followed by different letters are significantly different (Student’s *t*-test).

Parameter	Treated	Control
Height (m)	2.16 ± 0.17 a	1.90 ± 0.18 b
Number of leaves	22.13 ± 0.34 a	19.06 ± 0.34 b
First cob appearance (cm)	72.06 ± 1.01 a	66.5 ± 1.66 b
Number of cobs/plant	1.76 ± 0.11 a	1.2 ± 0.40 b
Number of nodes where the first cob appears	5.16 ± 0.13 a	4.36 ± 0.15 b
Number of grain rows/cob	14.3 ± 0.25 a	11.8 ± 0.28 b
Number of grains/row	34.5 ± 0.78 a	26.1 ± 1.2 b
Seed weight/plant (gr)	205.16 ± 3.2 a	98.8 ± 3.8 b
Yield (Kg/ha)	15,357.19 ± 35.8 a	7222.08 ± 38.7 b
Seed germination (%)	89 ± 2 a	77 ± 5.5 b
